# Cerebellum-inspired neural network solution of the inverse kinematics problem

**DOI:** 10.1007/s00422-015-0661-7

**Published:** 2015-10-05

**Authors:** Mitra Asadi-Eydivand, Mohammad Mehdi Ebadzadeh, Mehran Solati-Hashjin, Christian Darlot, Noor Azuan Abu Osman

**Affiliations:** Department of Biomedical Engineering, Faculty of Engineering, University of Malaya, 50603 Kuala Lumpur, Malaysia; Department of Computer Engineering and Information Technology, Amirkabir University of Technology, Tehran, 15914 Iran; Department of Biomedical Engineering, Amirkabir University of Technology, Tehran, 15914 Iran; Département de Traitement des signaux et des images, Ecole Nationale Supérieure des Télécommunications, 75634 Paris Cedex 13, France

**Keywords:** Inverse kinematics, Cerebellar neural network, Cerebellar cortex, Robot manipulator, Bioinspired model, Fuzzy neural network

## Abstract

The demand today for more complex robots that have manipulators with higher degrees of freedom is increasing because of technological advances. Obtaining the precise movement for a desired trajectory or a sequence of arm and positions requires the computation of the inverse kinematic (IK) function, which is a major problem in robotics. The solution of the IK problem leads robots to the precise position and orientation of their end-effector. We developed a bioinspired solution comparable with the cerebellar anatomy and function to solve the said problem. The proposed model is stable under all conditions merely by parameter determination, in contrast to recursive model-based solutions, which remain stable only under certain conditions. We modified the proposed model for the simple two-segmented arm to prove the feasibility of the model under a basic condition. A fuzzy neural network through its learning method was used to compute the parameters of the system. Simulation results show the practical feasibility and efficiency of the proposed model in robotics. The main advantage of the proposed model is its generalizability and potential use in any robot.

## Introduction

Robots are widely used primarily in industrial and medical applications where responsible, stable, and highly accurate operations are required. The demand today for more complex robots that have manipulators with higher degrees of freedom (Dof) is increasing because of technological advances. Obtaining the precise movement for a desired trajectory or sequence of arm and positions requires the computation of the inverse kinematic (IK) function, which is a major problem in robotics (Alavandar and Nigam 2008; Köker 2013; Wu and Rao 2007).

Forward kinematics involves determining the position of the end-effector of the robot given its joint variables. Obtaining the joint variable of a robot manipulator, given the desired position of the end-effector of the robot, is called IK (de Jesús Rubio et al. 2013; Zhang and Paul 1991). The solution of the IK problem requires the real-time computation and uniqueness of the inverse function. The solution of the IK problem has been studied by many researchers (Ali et al. 2010; Kanoun et al. 2011; Kumar et al. 2010; Reinhart and Steil 2011; Wang et al. 2010). Many approaches to solving the IK problem can be categorized into (1) analytical-based and (2) learning-based methods.

Although both types of methods can efficiently solve the problem, they have several shortcomings. First, the computations of the complex models in both methods are time-consuming because of the complexity of the mathematical formulation. Furthermore, poor efficiency results from the failure of the model to specify the robot characterization. By contrast, if the model can reflect all of the robot characterization, then the results are more specific. Second, the learning data generated from the IK function in a restricted domain in most learning-based methods result in limited convergence. Neither method proposes a general solution for IK problems (Hasan et al. 2006, 2010).

The proposed solution can be categorized into a learning-based method in the present study. However, two major distinctions can be recognized. First, this study proposes a new artificial neural network (ANN) model inspired by the anatomy of the cerebellum. Second, the IK function is learned using the forward kinematics function, unlike in other studies.

Different solutions to the IK problems of serial manipulators through learning-based methods exist. Among these solutions, learning-based methods that use ANNs are similar to the method used in the present study.

The approach used by Alavandar and Nigam (2008) in solving the IK problem is based on an adaptive neuro-fuzzy interface system, which was used to predict and estimate the problem. The said researchers collected training data from the forward kinematics of the 2-Dof and 3-Dof robot manipulator to show the effectiveness of this approach. The obtained results are inconsistent with the analytical IK function. The disadvantage of this method is its limited convergence because collecting data from forward kinematics covers only a part of the IK function domain.

Karlik and Aydin (2000) implemented a robot manipulator with six Dof through the best ANN configuration from two ANNs: a three-layer back-propagation (BP) with six outputs and six four-layer BPs with one output. The error obtained from the second ANN is smaller than that from the first configuration. The data set elements (i.e., inputs and outputs) for training NNs were calculated from analytic equations based on restrictions.

Xia and Wang (2001) developed a recurrent NN called the dual network, which has a single neuron layer. The approach proposed by Arefi and Sadigh (2011) is based on a fuzzy algorithm and a polar coordinate displacement of the robot manipulator’s tip. The model overcomes blind spots and singularities. NNs based on the radial basis function and multilayer perceptron, used to predict incremental joint angles, were proposed by Chiddarwar et al. and K.K. Dash et al., respectively (Chiddarwar and Ramesh Babu 2010; Dash et al. 2011). A genetic algorithm was used to improve the accuracy of the ANN model in several of these studies (Köker 2013; Oyama et al. 2001).

The ability of biological motor control systems to fine-tune themselves and enhance their performance is one of their major inspiring aspects. The cerebellum is indispensable in achieving fast and precise coordinated movements and accurately perceiving body motion; thus, the use of the cerebellum as a model for control and learning movement has attracted the attention of researchers for many years (Albus 1975b; Darlot 1993; Eccles et al. 1967; Kawato et al. 1987; Miall et al. 1993).

The current study attempts to modify the shortcomings of previous models of cerebellar NNs, which is more consistent with the general anatomy and functioning of the cerebellar pathways to solve the IK problem. Moreover, the disadvantage of previous cerebellar NN models and strong point of the proposed model have been mathematically proven.

## Method

### Cerebellar NN Model

Computing an inverse function is an “ill-posed problem” with no general solution, except in trivial cases (Cannon and Robinson 1987; Tikhonov and Arsenin 1979).

The forward kinematics function is shown in Eq. (1), where ${\varvec{{\uptheta }}} \mathbf{(t)} =(\theta _1 (t),\theta _2 (t), \ldots \theta _n (t))$ is the joint variable of a manipulator with *n* Dof at any instant of time. The position variable at any instant of time in the *x*, *y*, and *z* directions is denoted by $\mathbf{P(t)} =(x(t),y(t),z(t))$, and *f* is a nonlinear function.

1 $$\begin{aligned} \mathbf{P(t)} =f({\varvec{{\uptheta }}} \mathbf{(t)}) \end{aligned}$$

By contrast, the IK function can be computed using Eq. (2). However, the inverse function $(f^{-1})$ is not unique, and the set of solution is infinite because of the nonlinear, uncertain, and time-varying nature of *f*.

2 $$\begin{aligned} {\varvec{{\uptheta }}} (\mathbf{t})=f^{-1}(\mathbf{P}(\mathbf{t})) \end{aligned}$$

A direct function is deterministic, whereas an inverse function is not necessarily so. Therefore, similar effects can be induced by different sets of causes. For instance, the biomechanical function of the arm, which expresses movement caused by exerting forces, is deterministic according to Newton’s law. Conversely, the same hand displacement can result from various configurations of articulated arm segments and can be induced at different levels of stiffness of articulations. Given that different causes can produce similar effects, a cause–effect relationship is generally not bijective but is rather a surjection from the domain of the causes to that of the effects. Therefore, no general method permits a definite return from an effect to a single cause, and finding an inverse function is a process that is very sensitive to the initial conditions and noise. Thus, in practice, an inverse function that is appropriate at one instant can be inappropriate at the next. Specifically, the number of possible solutions is infinite for the IK of a limb, when the number of moving segments is larger than the Dof of the end-effectors. Similarly, an infinite number of solutions are possible for inverse dynamics, when the number of actuators (muscles) is larger than that of moving segments.

One of the early famous computational models of cerebellum is the cerebellar model articulation controller, which is based on the Marr–Albus ideas of the cerebellum (Albus 1975a, b; Pouget et al. 2000; Wolpert 1997). However, it was not originally proposed as a biological plausible approximation (Wolpert et al. 1998).

Given the unavailability of a training signal to the central nervous system (CNS), one of the most challenging tasks in modeling the cerebellum as an inverse model is acquiring an inverse dynamic model through motor learning. Kawato and colleagues (Kawato et al. 1987; Kawato and Gomi 1992) proposed a cerebellar feedback-error- learning model to solve this problem.

The learning process in the Kawato model is conducted in slow movement, after which the speed is increased . However, the model is insensitive to the noise because of the use of an open-loop control system, but one of the most important shortcomings is that it is not based on cerebellum physiology. Previous studies show that the cerebellum does not compute the inverse dynamic solutions and instead learns the forward one (Gentili et al. 2009).

The other inverse model for the cerebellum as a controller is based on the Smith predictor (Miall et al. 1993). This model is a forward model and has a delay structure that postpones the sensorimotor predictions for regulating the sensorimotor outcomes. In contrast to the Kawato model, the Smith model uses the inverse function, and the main (forward) function is trained. One of its advantages is that the error in this model is lower than that in the Kawato model (Wolpert et al. 1998).

The inverse problem can be bypassed by placing in a feedback loop a circuit that predicts the effect of motor orders, which provides it with a deterministic direct function (Barto et al. 1999; Darlot 1993; Houk 1996; Miall 1998; Miall et al. 1993; Miall and Wolpert 1996; Wolpert et al. 1998). The proposed theory holds that anticipating signal values is similar to the function of the cerebellar cortex and that computing approximate inverse functions is similar to the function of the entire cerebellum.

**Fig. 1:**
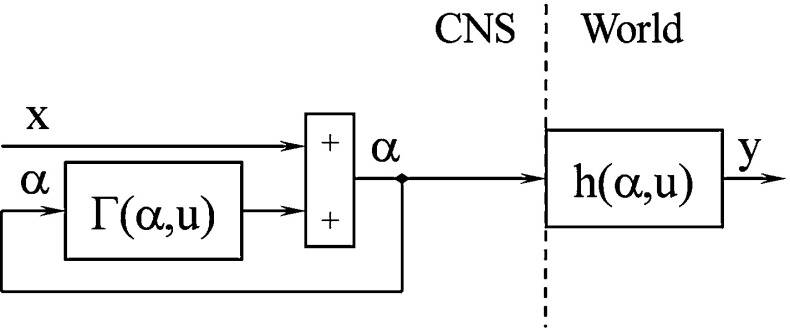
Feedback model of a cerebellar cortex (Ebadzadeh and Darlot 2003; Ebadzadeh et al. 2005)

The model in Fig. 1 shows a feedback loop with a direct function ${\varGamma }(\alpha ,\, u)$, a short delay of the feedback loop, and a delay to the motoneuron $(\alpha )$. The mathematical equations used to obtain ${\varGamma } (\alpha ,\, u)$ with $x(t)=y(t) \quad x=y$ are as follows [i.e., *x*(*t*) is the input signal and *y*(*t*) is the output]:

3 $$\begin{aligned}&y(t) = h(\alpha (t),u(t)) \end{aligned}$$

4 $$\begin{aligned}&\alpha (t+1) = x(t)+\Gamma (\alpha (t),u(t)) \end{aligned}$$

This equation must be $\alpha (t+1)=\alpha (t)$ to obtain a fixed point.

Thus,

5 $$\begin{aligned} x(t)=\alpha (t)-\Gamma (\alpha (t),u(t)) \end{aligned}$$

With $x=y$ and (3) and (5) combined,

6 $$\begin{aligned} x(t)=y(t)\Rightarrow \Gamma (\alpha (t),u(t))=\alpha (t)-h(\alpha (t),u(t)) \end{aligned}$$

Substituting (6) into (4) yields

7 $$\begin{aligned} \alpha (t+1)=g(\alpha (t))=\alpha (t)+x(t)-h(\alpha (t),u(t)) \end{aligned}$$

**Fig. 2:**
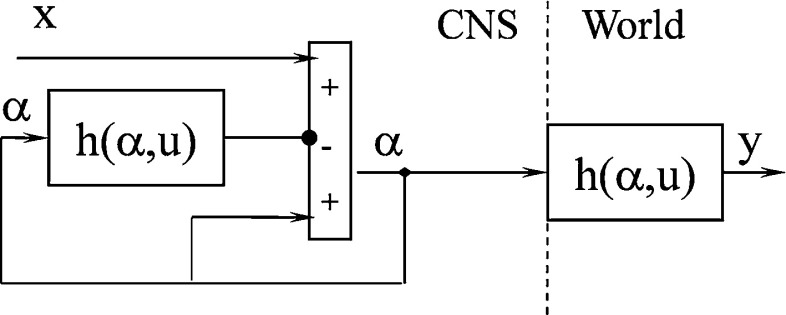
Modified model of a cerebellar cortex (Ebadzadeh et al. 2005; Gentili et al. 2009)

The modified model with respect to the equation for obtaining a fixed point is shown in Fig. 2.

**Fig. 3:**
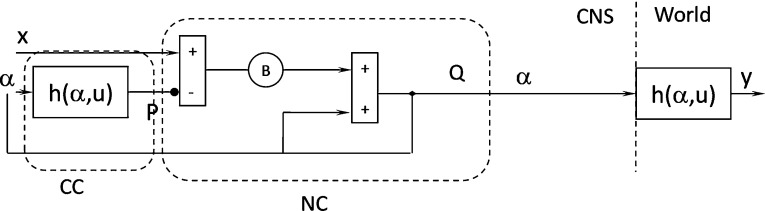
Proposed model of a cerebellar cortex, where CC is the cerebellar cortex and NC is the cerebellar nucleus

The condition of the model stability (proven in “Appendix 1”) is

8 $$\begin{aligned} 0<\frac{\partial h}{\partial \alpha }<2 \end{aligned}$$

The model is sometimes unstable because this condition is unsatisfied in every situation. The following modified model (Fig. 3), however, can satisfy the condition in every situation:

9 $$\begin{aligned}&y(t) = h(\alpha (t),u(t)) \end{aligned}$$

10 $$\begin{aligned}&\alpha (t+1) = g(\alpha (t))=\alpha (t)+B\left[ {x(t)-h(\alpha (t),u(t))} \right] \nonumber \\ \end{aligned}$$

The above equation must be $\alpha (t+1)=\alpha (t)$ to obtain a fixed point.

Thus,

11 $$\begin{aligned} B\left[ {x\left( t \right) -h\left( {\alpha \left( t \right) ,u\left( t \right) } \right) } \right] =0 \end{aligned}$$

and

12 $$\begin{aligned} x\left( t \right) =h\left( {\alpha \left( t \right) ,u\left( t \right) } \right) \end{aligned}$$

Combining (9) and (12) yields $\Rightarrow x(t)=y(t)$.

Therefore, the proposed model works as an inverter. The stability condition of the proposed model (calculated in “Appendix 2”) is shown in (22):

13 $$\begin{aligned} 0<B\frac{\partial g}{\partial \alpha }<2 \end{aligned}$$

Thus, the proposed model works in every situation because *B* can be varied as the stability condition is satisfied.

**Fig. 4:**
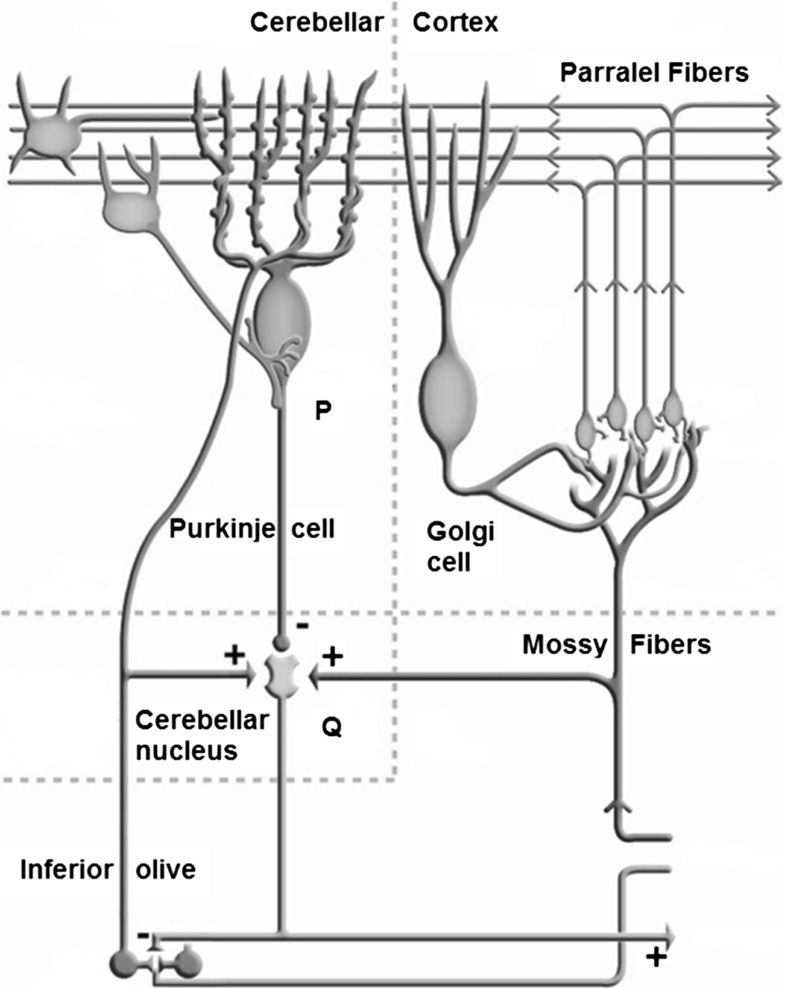
Anatomy schematic diagram of human cerebellar cortex (Eccles et al. 1967; Kandel et al. 2000)

This circuit resembles the cerebellar pathways because positive and negative loops arranged in parallel evoke the excitatory mossy fibers that reach the cerebellar nuclei and cerebellar cortex. The inhibitory axons of the Purkinje cells project to the cerebellar nuclei. Thus, the predictor resembles the cerebellar cortex, and the summing element resembles the cerebellar nuclei (Figs. 3, 4). This anatomical interpretation matches the importance of the cerebellum in motor coordination and error compensation. The cerebellum is indispensable in achieving fast and precise coordinated movements and accurately perceiving body motion.

The input signal *x* reaches the summing element, which issues $\alpha$ through two pathways: a direct pathway that transmits this signal unchanged and an indirect pathway that processes the signal. This side circuit represents a motor part of the cerebellar pathways, which are situated outside the direct sensory or motor pathways.

The element, $h\, (\alpha ,\, u)$, which is the predictor, represents the cerebellar cortex, which receives many sensory signals and efferent copies of motor signals through the mossy fibers. The cerebellar cortex processes these signals to increase the activity of Purkinje cells. This activity *P* is assumed to encode a dynamic signal that predicts the state of the body at the time the motor orders generate their effects (Figs. 3, 4).

The summing element placed immediately downstream $h \, (\alpha ,\, u)$, where the signal of the positive loop is summed to the output *P* of $h \, (\alpha ,\, u)$ and issues the signal *Q*, represents a group of neurons in a cerebellar nucleus (Figs. 3, 4). The same feedback signals reach the predictor and summing element, as the messages conveyed by the mossy fibers reach both the cerebellar cortex and cerebellar nuclei. The negative output of the predictor is comparable with the inhibitory projection of the Purkinje cells to the neurons of the cerebellar nuclei (Figs. 3, 4).

The cerebellum has three learning levels: (1) unsupervised learning in the glomerulus synapse, (2) supervised learning in the Purkinje synapse, and (3) supervised learning in the cerebellar nucleus (Jaberi et al. 2013; Jaeger 2013). The first and second learning methods have been previously reported (Bostan et al. 2013; Ebadzadeh et al. 2005; Schweighofer et al. 2013).

### Fuzzy NN (FNN)

Calculating the optimal value of the model’s parameter (*B* in Eq. 13) is computationally complex. ANN must be employed to predict this value at any instant of time. FNN is used in this study to predict the optimal value of the model’s parameter at any instant of time. The other ANNs that work as predictor cannot be employed because the nonlinear function (i.e., feedback loop in the model) learning methods can be implemented only in the FNNs.

The approximation of nonlinear functions can be modeled using a fuzzy rule based on a set of if–then rules defined as follows:

14 $$\begin{aligned} R^{i}=\hbox {if}\, x_1 \hbox { is } A_1^i \hbox { and } \ldots \hbox { and } x_n \hbox { is } A_n^i \hbox { then } y \hbox { is } B^{i} \end{aligned}$$

where $$A_j^i $$ and $$B^{i}$$ are fuzzy sets, $$x=(x_1 , \ldots ,x_n)^\mathrm{T}$$ is the input variable, and *y* is the output variable of the fuzzy system.

Equation (15) is used to map a fuzzy set ${A}'$ to a fuzzy set ${B}'$ through the product inference engine.

15 $$\begin{aligned} \mu _{{B}'} (y)=\mathop {\max }\limits _{i=1}^m \left[ \sup \left( \mu _{{A}'} (x)\prod _{j=1}^n \mu _{A_j^i} \left( {x_j} \right) \mu _{B^{i}} (y)\right) \right] . \end{aligned}$$

A real-value point $x^{*}$ can be fuzzified by a singleton fuzzifier, which maps $x^{*}$ to a fuzzy singleton ${A}'$ with a membership value of 1 at $x^{*}$ and 0 at other points:

16 $$\begin{aligned} \mu _{{A}{'}}{}^{\prime }(x)= \left\{ \begin{array}{ll} 1 &{} \quad x=x^{*} \\ 0 &{} \quad \hbox {otherwise} \\ \end{array} \right. \, \end{aligned}$$

A defuzzifier is an algorithm that maps from a fuzzy set *B* to a crisp point $$y^{*}$$. $$y^{-i}$$ is the center of the *i*th fuzzy set and $$w_i $$ is its height. The center average defuzzifier determines $$y^{*}$$ as

17 $$\begin{aligned} y^{*}=\frac{\sum _{i=1}^m {y^{-i}w_i } }{\sum _{i=1}^m {w_i } } \end{aligned}$$

**Fig. 5:**
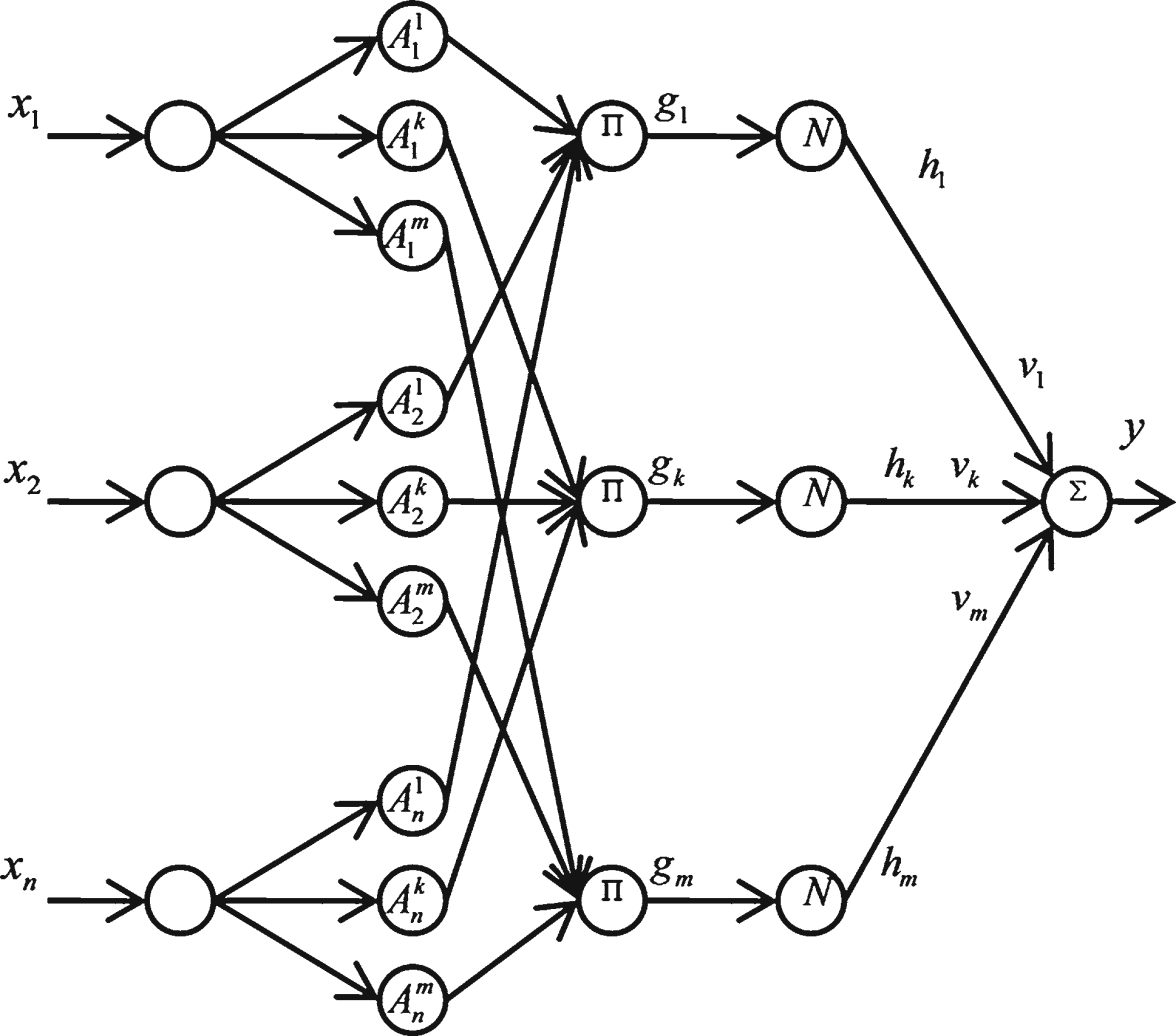
Fuzzy neural network structure

Fuzzy systems with the fuzzy rule base, a product inference engine, a singleton fuzzifier, and a center average defuzzifier have the following form for a fuzzy set $B^{i}$ with a center$y^{-i}$:

18 $$\begin{aligned} f(x)=\frac{\sum _{i=1}^m {y^{-i}\prod _{j=1}^n {\mu _{A_j^i } (x_j )} } }{\sum _{i=1}^m {\prod _{j=1}^n {\mu _{A_j^i } (x_j )} } } \end{aligned}$$

where *x* and *f*(*x*) are the input and output of the fuzzy system (Wang 1999). The corresponding network of the fuzzy model can be built as shown in Fig. 5. The FNN has four layers. The output of each node in the first layer equals $\mu _{A_j^i } (x_j)$ and the membership value of fuzzy set $A_j^i$ . The nodes in the second layer are used to calculate the product of the membership values and inputs in all the dimensions for each rule:

19 $$\begin{aligned} g_i =\prod _{j=1}^n {\mu _{A_j^i } (x_j).} \end{aligned}$$

The third layer is the normalization layer, where the output of node *i* is calculated as follows:

20 $$\begin{aligned} h_i =\frac{g_i }{\sum _{i=1}^m {g_i } } \end{aligned}$$

Finally, the output of the *i*th node of the last (output) layer is the summation of its input values from the previous layer:

21 $$\begin{aligned} y=\sum _{i=1}^M {v_i h_i } , \end{aligned}$$

where $v_i$ s are the consequent parameters that should be learned through least-squares or gradient descent. For the Takagi–Sugeno–Kang fuzzy model, one layer before the output layer is added to replace the consequent parameters with a linear combination of inputs. Thus, the output of the network is calculated as follows:

22 $$\begin{aligned} y=\sum _{i=1}^M {\left( c_0^i +c_1^i x_1 + \cdots +c_n^i x_n \right) } h_i . \end{aligned}$$

A hybrid learning algorithm determines the parameters in several neuro-fuzzy networks, wherein epochs involve forward and backward passes. All training data are presented to the network, and output weights are identified by the least-squares algorithm in the forward pass. Recursive least squares can also be used to determine the weights of the output layers (Kosko 1994; Malek et al. 2012).

**Fig. 6:**
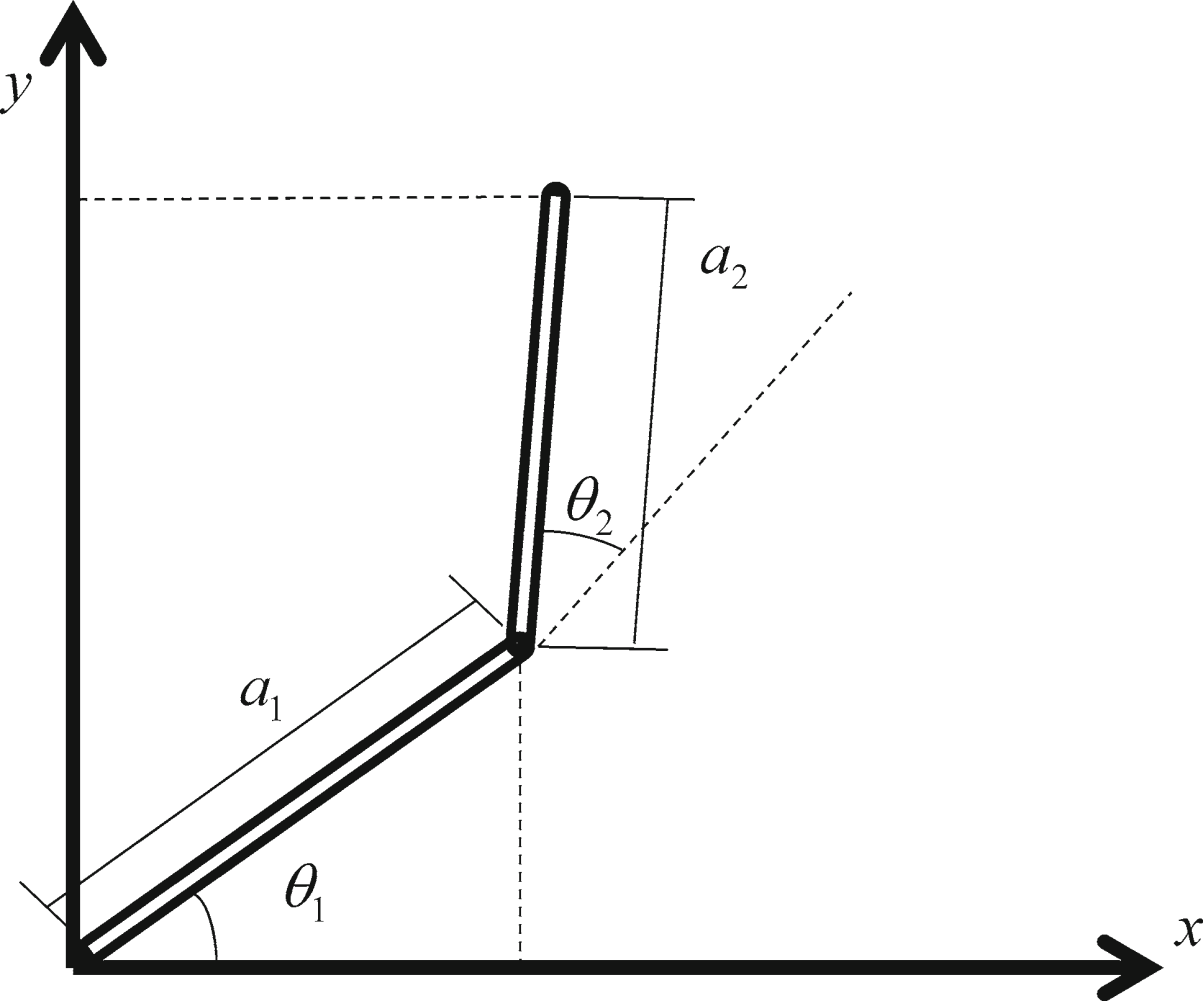
Forward kinematics of the two-segmented arm

**Fig. 7:**
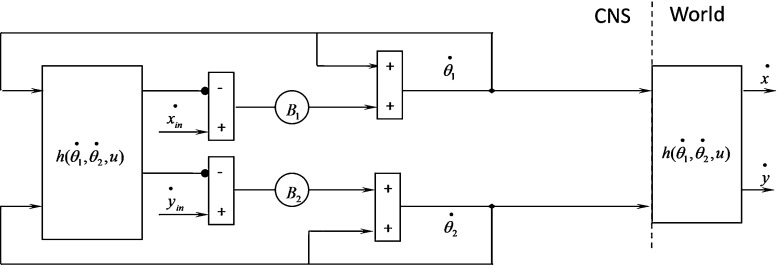
Developed cerebellar cortex model for solving two-segmented arm IK

### Proposed model to solve the IK problem

The control of the two-segmented arm is studied to test the proposed model. This examination requires the calculation of IK with two inputs and two outputs. The model in the previous section is proposed with one input and one output, so that the proposed model must be developed. In this section, the equation of the forward kinematics of the two segments is calculated, and the proposed model is developed.

With regard to Fig. 6, the equation of forward kinematics can be written as follows:

23 $$\begin{aligned} x= & {} a_1 \cos \theta _1 +a_2 \cos (\theta _1 +\theta _2 ) \end{aligned}$$

24 $$\begin{aligned} y= & {} a_1 \sin \theta _1 +a_2 \sin (\theta _1 +\theta _2 ) \end{aligned}$$

where $a_1$ and $a_2$ are the lengths of the first and second segments, $\theta _1$ and $\theta _2$ are the angles of the same segments, and *x* and *y* are the Cartesian positions of the end-effector.

The velocity of *x* and *y* at any instant of time is calculated as follows:

25 $$\begin{aligned} \mathop x\limits ^{\bullet }= & {} -a_1 \sin \theta _1 \mathop {\theta _1 }\limits ^{\bullet } -a_2 \sin (\theta _1 +\theta _2 )(\mathop {\theta _1 }\limits ^{\bullet } \mathop {+\mathop {\theta _2 }\limits ^{\bullet }}) \end{aligned}$$

26 $$\begin{aligned} \mathop y\limits ^{\bullet }= & {} a_1 \cos \theta _1 \mathop {\theta _1 }\limits ^{\bullet } +a_2 \cos (\theta _1 +\theta _2 )(\mathop {\theta _1 }\limits ^{\bullet } \mathop {+\mathop {\theta _2 }\limits ^{\bullet }}) \end{aligned}$$

The developed model with regard to the previous equations for controlling a two-segmented arm is shown in Fig. 7.

27 $$\begin{aligned} \mathop {\theta _1 }\limits ^{\bullet } (t+1)= & {} g_1 (\mathop {\theta _1 }\limits ^{\bullet } (t),\mathop {\theta _2 }\limits ^{\bullet } (t)) \nonumber \\= & {} \mathop {\theta _1 }\limits ^{\bullet } (t)+B_1 (\mathop x\limits ^{\bullet } (t)-\mathop {x}\limits ^{\bullet }{}_{in} (t)) \end{aligned}$$

28 $$\begin{aligned} \mathop {\theta _2} \limits ^{\bullet } (t+1)= & {} g_2 (\mathop {\theta _1 }\limits ^{\bullet } (t),\mathop {\theta _2 }\limits ^{\bullet } (t)) \nonumber \\= & {} \mathop {\theta _2 }\limits ^{\bullet } (t)+B_2 (\mathop y\limits ^{\bullet } (t)-\mathop {y}\limits ^{\bullet }{}_{in} (t)) \end{aligned}$$

The equations must be $\mathop {\theta _1 }\limits ^{\bullet } (t+1)=\mathop {\theta _1 }\limits ^{\bullet } (t)$ and $\mathop {\theta _2 }\limits ^{\bullet } (t+1)=\mathop {\theta _2}\limits ^{\bullet } (t)$ to obtain a fixed point.

Thus,

29 $$\begin{aligned} B_1 (\mathop x\limits ^{\bullet } (t)-\mathop {x}\limits ^{\bullet }{}_{in} (t))= & {} 0 \qquad \Rightarrow \qquad \mathop x\limits ^{\bullet } (t)=\mathop {x}\limits ^{\bullet }{}_{in} (t) \end{aligned}$$

30 $$\begin{aligned} B_2 (\mathop y\limits ^{\bullet } \left( t \right) -\mathop {y}\limits ^{\bullet }{}_{in} (t))= & {} 0 \qquad \Rightarrow \qquad \mathop y\limits ^{\bullet } (t)=\mathop {y_{in} (t)}\limits ^{\bullet } \end{aligned}$$

The results verify that the developed model acts as an inverter.

**Fig. 8:**
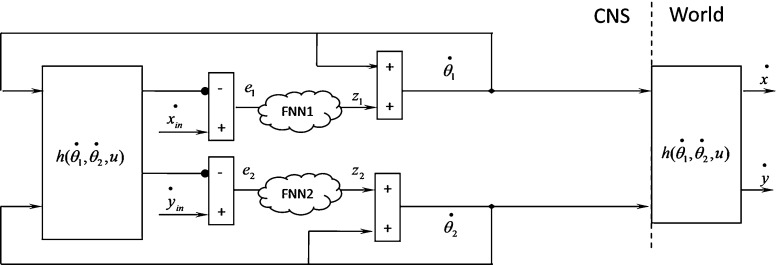
Using FNN in the developed cerebellar cortex model

The stability condition of the developed model (calculated in the “Appendix 3”) is shown in Eq. (31):

31 $$\begin{aligned} -4< & {} \left( B_1 \frac{\partial \mathop x\limits ^{\bullet } }{\partial \mathop {\theta _1 }\limits ^{\bullet } }+B_2 \frac{\partial \mathop y\limits ^{\bullet } }{\partial \mathop {\theta _2 }\limits ^{\bullet } }\right) \nonumber \\&\quad \pm \sqrt{\left( B_1 \frac{\partial \mathop x\limits ^{\bullet } }{\partial \mathop {\theta _1 }\limits ^{\bullet } }+B_2 \frac{\partial \mathop y\limits ^{\bullet } }{\partial \mathop {\theta _2 }\limits ^{\bullet } }\right) ^{2}+4B_1 B_2 \frac{\partial \mathop x\limits ^{\bullet } }{\partial \mathop {\theta _2 }\limits ^{\bullet } }\frac{\partial \mathop y\limits ^{\bullet } }{\partial \mathop {\theta _1 }\limits ^{\bullet } }}<0\nonumber \\ \end{aligned}$$

**Fig. 9:**
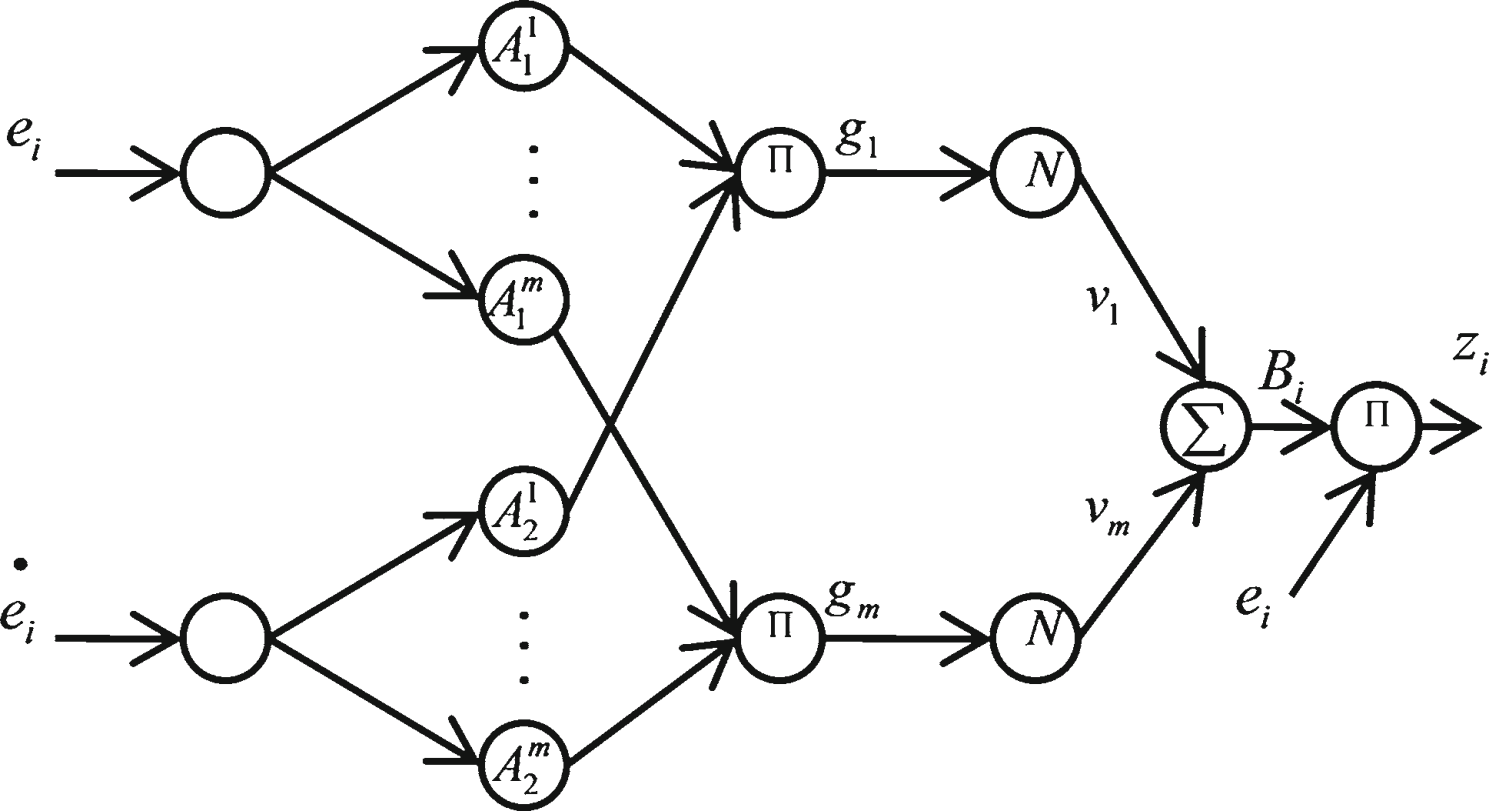
The structure of FNN used in the model

**Fig. 10:**
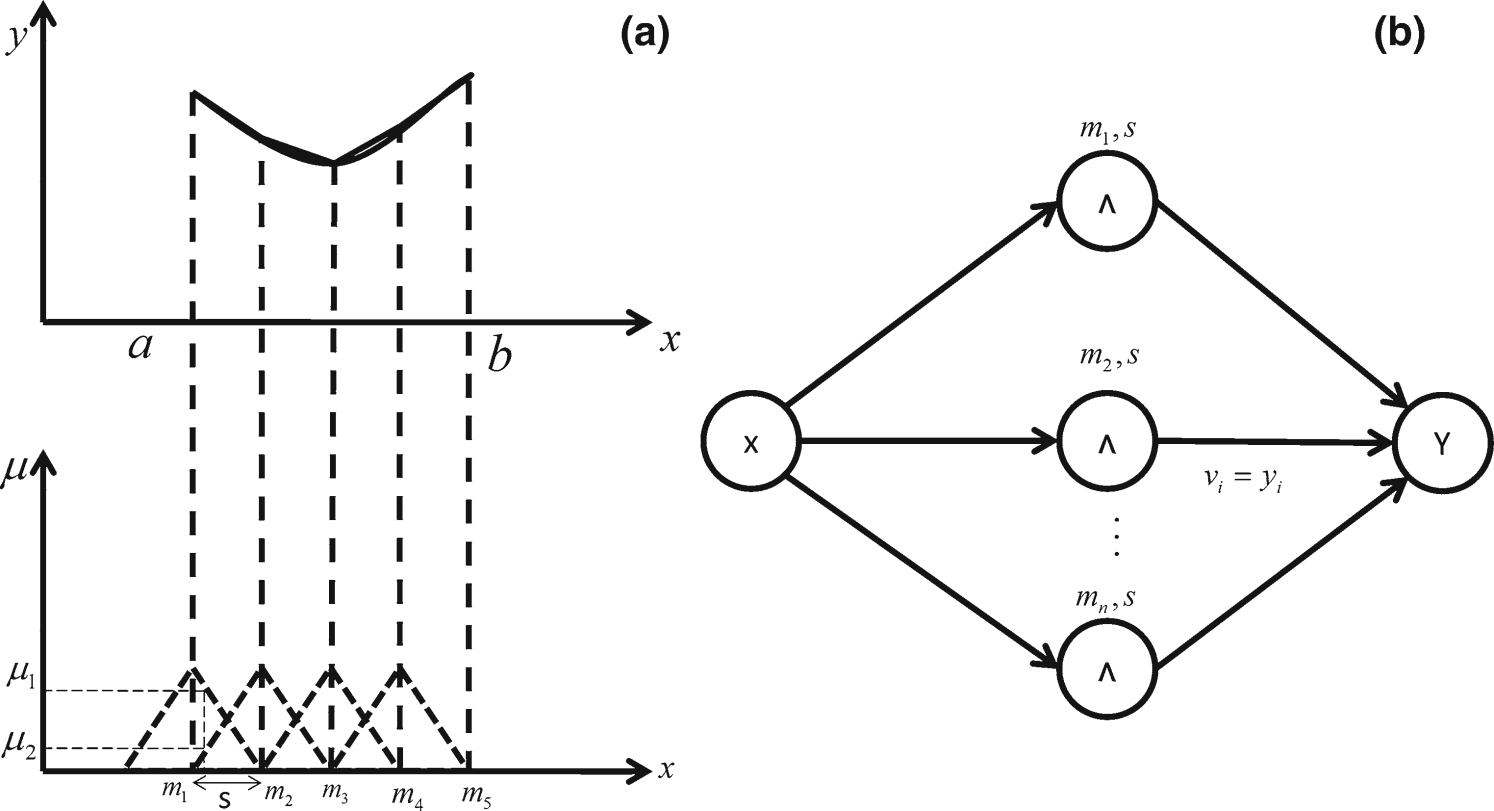
**a** Normal triangular fuzzy sets and **b** rules for learning the weights of the network

The coefficients $$B_1 $$ and $$B_2$$ must satisfy the condition of Eq. (31) to prove the stability of the proposed model. The values of these coefficients depend on Eqs. (25) and (26). The appropriate values of $$B_1 $$ and $$B_2$$ depend on angles $$\theta _1 $$ and $$\theta _2 $$ and their velocity at any instant of time, which indicates that the optimal values of $$B_1 $$ and $$B_2 $$ are dynamic and different at any instant of time. This computational complexity necessitates the calculation of optimal values with an FNN.

**Fig. 11:**
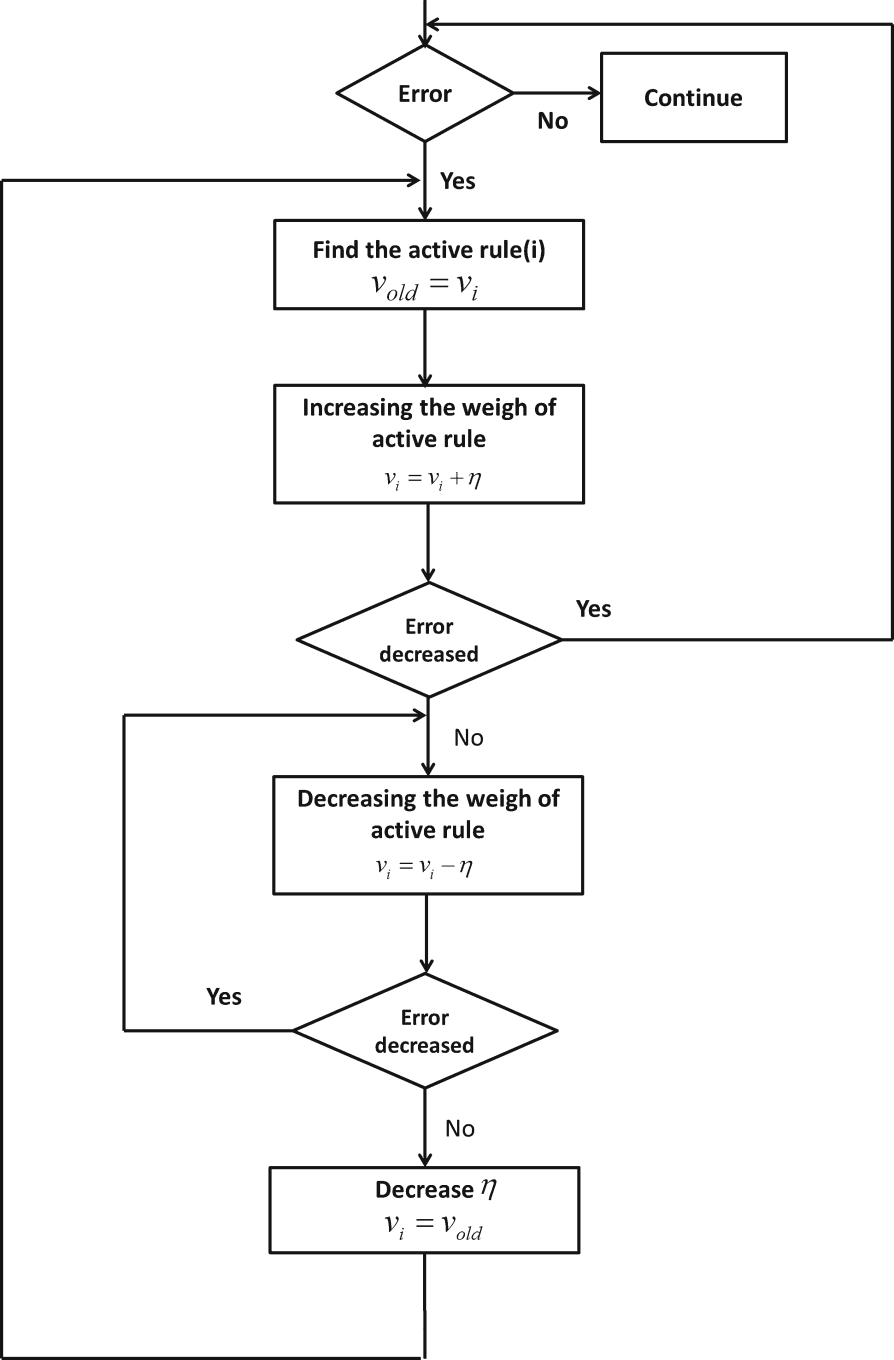
Flowchart of learning algorithm used for learning the weights of FNN

Figure 8 shows the location of two FNNs, and Fig. 9 shows the proposed FNN for learning $$B_1 $$ and $$B_2 $$.

The function $h=(\mathop {\theta _1}\limits ^{\bullet } ,\mathop {\theta _2} \limits ^{\bullet }, u)$ is unknown, so that the weights $v_i$ of the network must be learned. One of the most important reasons for selecting FNNs is the learning method, which runs only through these networks. The FNN used in this study is a one-layer network with a normal triangular fuzzy set. Normal triangular fuzzy sets are created as shown in Fig. 10, where $\mu$ and *v* are the membership function and weight, respectively.

Considering $y=f(x)$, the output of the network is calculated according to $y=\sum {v_i \mu _{A_i}}(x)$. If the condition $v_i =f(m_i)$ is true, then *f*(*x*) can be defined as a piecewise linear function. Given that *f*(*x*) is an unknown function, the weight $v_i$ should be learned by the network. Gradient and pseudo-inverse learning methods are inapplicable because the functions in the proposed model are nonlinear and cannot be differentiated as a result of the feedback loop.

The FNN has several features that make it an appropriate object of learning through the proposed learning method:

1. The point values in the fuzzy sets equal the function $(\mu =1)$.
2. The points in the *s* intervals are a linear combination of the preceding and following output values $(y=\mu _1 y_1 +\mu _2 y_2 )$.
3. The fuzzy sets in this study are normal; hence, $\mu _1 +\mu _2 =1$.

Therefore, only a maximum of two rules is equal to one, and the other rules are equal to zero at every instant of time.

We use FNN in this study because the nodes in the other networks are active at each step, which entails high cost. Our solution to this problem is shown as a learning algorithm in Fig. 11.

**Fig. 12:**
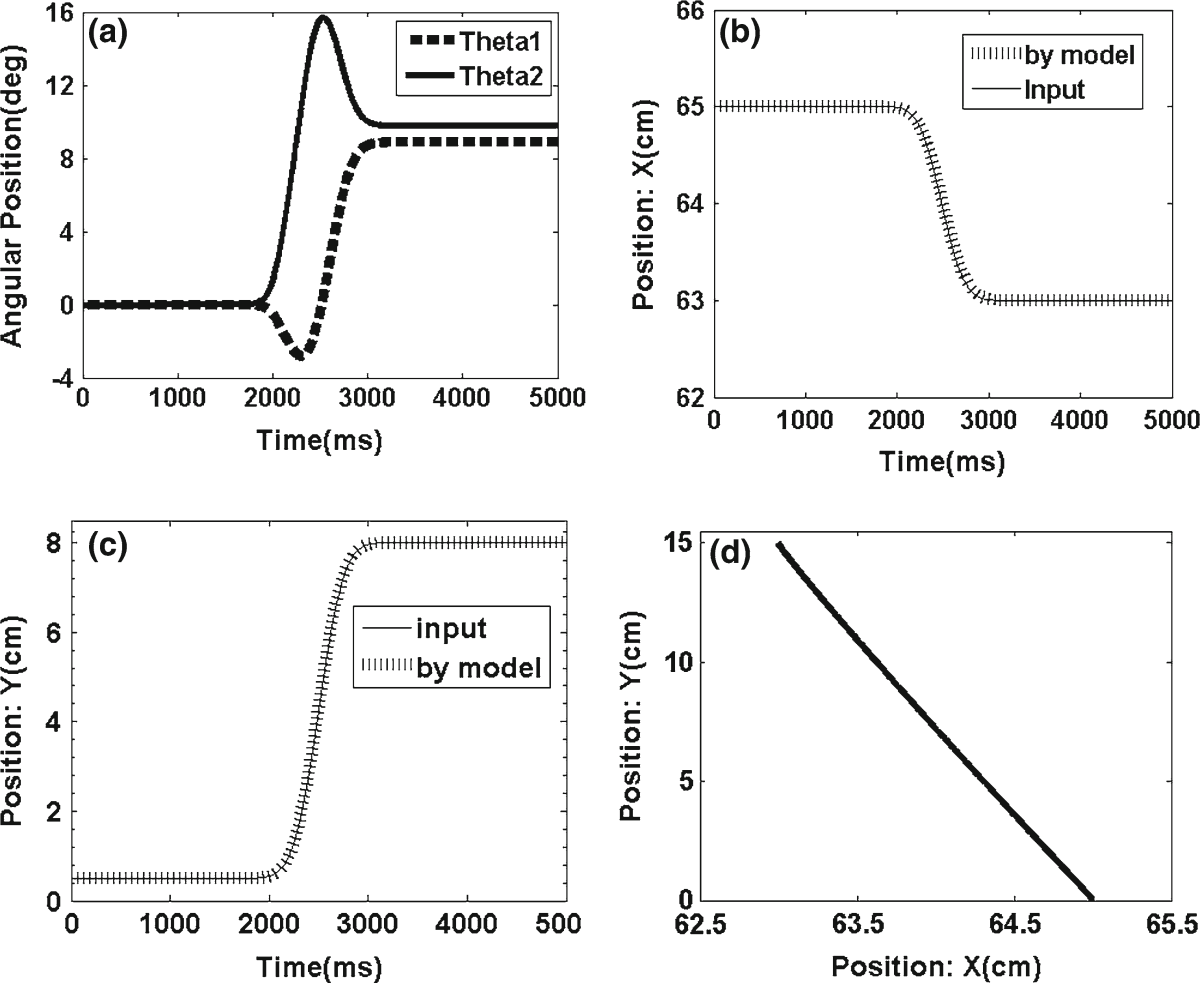
Movement profile of the two-segmented arm from point (65,0) to point (63,15). **a** Angles of the two-segmented arm that reach the desire point. **b** Arm displacement in the *x* axis direction from point 65 to point 63. **c** Arm displacement in the *y* axis direction from point 0 to point 15. **d** Trajectory in the joint coordinates

As previously mentioned, we cannot use differentiation because of the feedback loop. Therefore, whether or not the weight of the active rule at each step must be increased or decreased cannot be determined. A solution to this problem is to increase the weight of the active rule and subsequently calculate the error (Fig. 11). If the error decreases, the increasing step is continued until the error becomes constant or drops. If the error increases, the weight must be decreased until the error is stabilized.

## Results and discussion

Three experiments are designed and implemented in MATLAB (R2008a) 7.6.0.324 to evaluate the proposed model. The lengths of the first and second segments are considered to be $a_1 =0.35 \hbox { m}$ and $a_2 =0.3 \hbox { m}$, respectively. The Gaussian function considers the velocity of $\theta$ to be the input of the model, and the velocity of movement is considered to be $\sigma =0.2$.

**Fig. 13:**
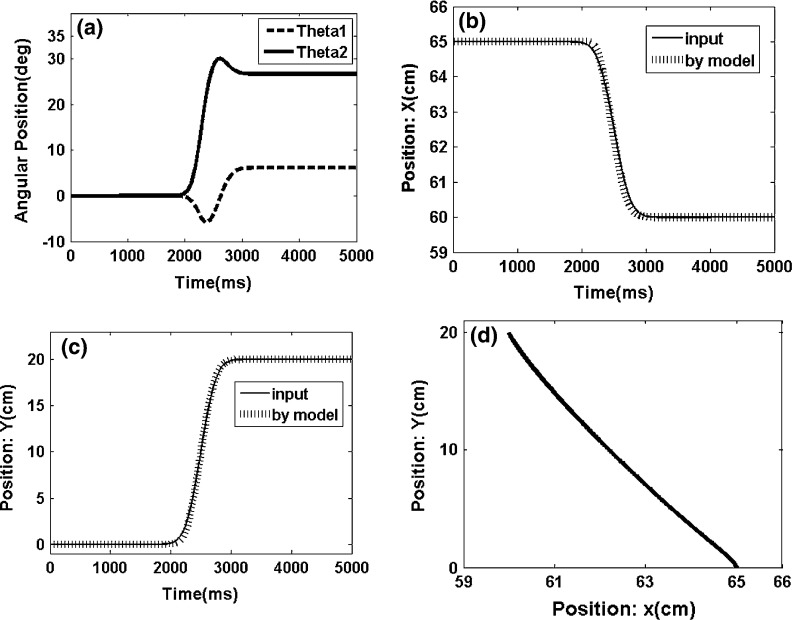
Movement profile of the two-segmented arm from point (65,0) to point (60,20). **a** Angles of the two-segmented arm that reached the desired point. **b** Arm displacement in the *x* axis direction from point 65 to point 60. **c** Arm displacement in the *y* axis direction from point 0 to point 20. **d** Trajectory in the joint coordinates

**Fig. 14:**
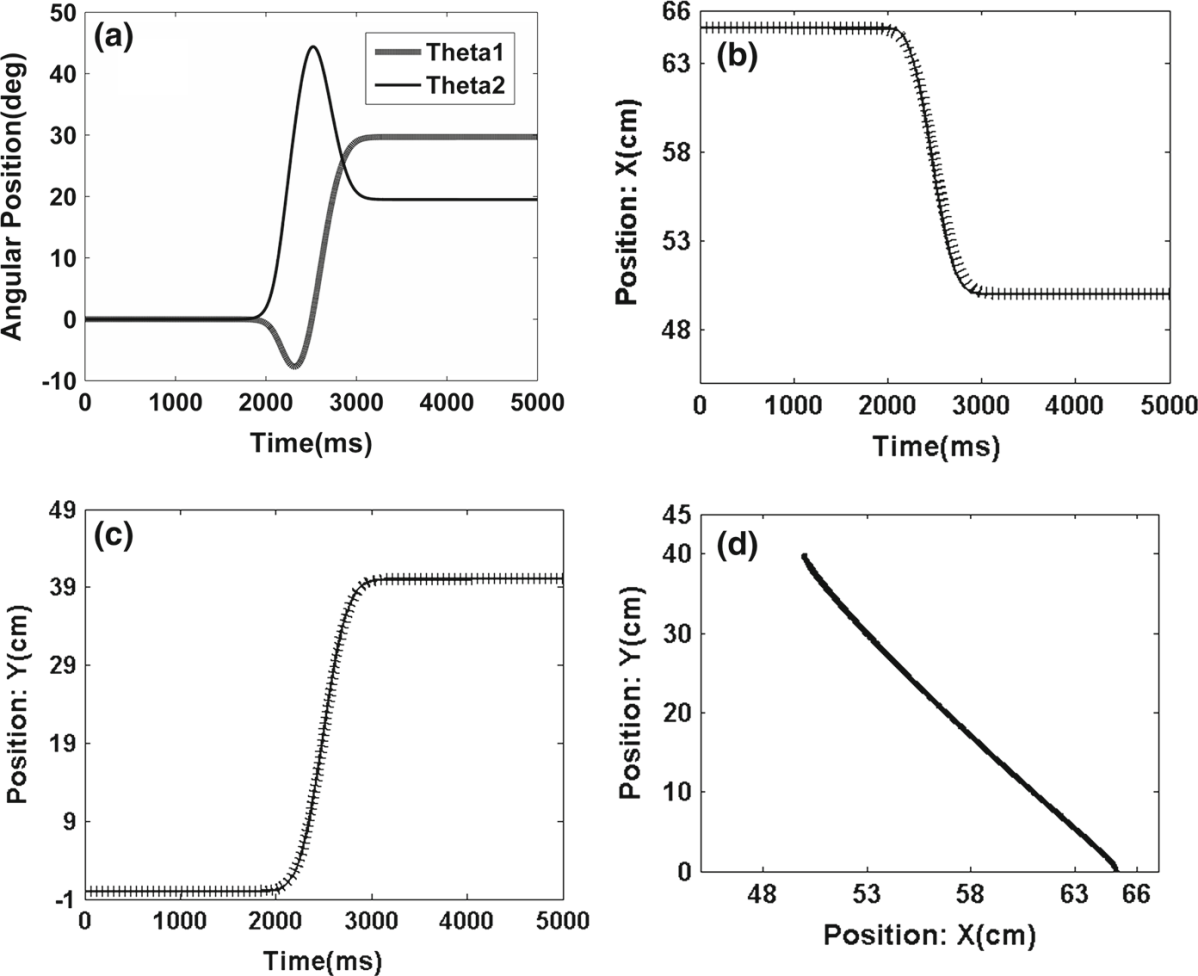
Movement profile of the two-segmented arm from point (65,0) to point (50,40). **a** Angles of the of the two-segmented arm that reach the desired point. **b** Arm displacement in the *x* axis direction from point 65 to point 50. **c** Arm displacement in the *y* axis direction from point 0 to point 40. **d** Trajectory in the joint coordinates

The end-effector of the two-segmented robot moves from point (65, 0) to point (63, 15) in the first experiment (Fig. 12). The movement exhibits a trajectory in the joint coordinates, which is expected to be a straight line in the best-case scenario. Figures 12a, 13 and 14a show the angles of each arm segment that reach the desired position.

The end-effector of the two-segmented robot moves from point (65,0) to point (60,20) and from point (65,0) to point (50,40) in the second and third experiments, respectively (Figs. 13, 14).

The models provide satisfying results, and the trajectory in the joint coordinates in all three experiments is approximately a straight line (Figs. 13, 14). These results show that the error of the model is acceptable in all cases (i.e., the mean square error is below the 0.0001).

Thus, the results of experiments show that the proposed approach is a feasible option for the real-time path planning and precise control of robots.

## Conclusion

The IK problem is solved through a method that resembles cerebellar anatomy and function. Previous cerebellum-inspired solutions were based on a direct function and a recursive model that remains steady only under certain conditions. Therefore, they cannot be applied to robot movement with high Dof.

We used mathematical analysis to propose a modified model that is stable under all conditions because only one parameter is determined. This dynamic parameter varies at any instant. Therefore, we use an FNN with a particular learning method.

The proposed model is developed and modified for a simple two-segmented arm to show its feasibility. Two model parameters are approximated by FNN because of its specific learning method. The model has an acceptable and reliable performance in solving the IK problem of the two-segmented arm.

Moreover, the proposed model can be generalized for all functions because it is independent of the calculations for the inverse function, which is proven mathematically. This approach can be used for the prediction of IK solutions for any kind of robot regardless of the geometry and Dof associated with it.

## Appendix 1

There are some methods obtaining the model stability. One of them is to linearize it in its fixed point.

The linear equation of $g(\alpha (t))$ in $\alpha _0$ is as follows:

32 $$\begin{aligned} g(\alpha (t))-g(\alpha _0 )=\left. {\frac{\partial g}{\partial \alpha }} \right| _{\alpha _0 } (\alpha (t)-\alpha _0) \end{aligned}$$

With consideration of Eq. (16),

33 $$\begin{aligned} g(\alpha (t))=\alpha (t+1) \end{aligned}$$

And because $\alpha _0$ is fixed point of $g(\alpha (t))$,

34 $$\begin{aligned} g(\alpha _0)=\alpha _0 \end{aligned}$$

With (32), (33), and (34) combined,

35 $$\begin{aligned} \alpha (t+1)-\alpha _0 =\left. {\frac{\partial g}{\partial \alpha }} \right| _{\alpha _0 } (\alpha (t)-\alpha _0) \end{aligned}$$

And with replacement $t+1$ in (35),

36 $$\begin{aligned} \alpha (t+2)-\alpha _0 =\left. {\frac{\partial g}{\partial \alpha }} \right| _{\alpha _0 } (\alpha (t+1)-\alpha _0) \end{aligned}$$

With (35) and (36) combined,

37 $$\begin{aligned} \alpha (t+2)-\alpha _0 = \left( \left. \frac{\partial g}{\partial \alpha } \right| _{\alpha _0} \right) ^{2}(\alpha (t)-\alpha _0) \end{aligned}$$

Thus,

38 $$\begin{aligned} \alpha (t+n)-\alpha _0 =\left( {\left. {\frac{\partial g}{\partial \alpha }} \right| _{\alpha _0 } } \right) ^{n}(\alpha (t)-\alpha _0) \end{aligned}$$

For stability must be:

39 $$\begin{aligned} \alpha (t+n)=\alpha _0 \end{aligned}$$

This means that

40 $$\begin{aligned} -1<\left. {\frac{\partial g}{\partial \alpha }} \right| _{\alpha _0 } <1 \end{aligned}$$

With derivation of Eq. (16),

41 $$\begin{aligned} \frac{\partial g}{\partial \alpha }=1-\frac{\partial h}{\partial \alpha } \end{aligned}$$

With (40) and (41) combined,

42 $$\begin{aligned} 0<\frac{\partial h}{\partial \alpha }<2 \end{aligned}$$

## Appendix 2

The linear equation of $g(\alpha (t))$ in $\alpha _0$ is as follows:

43 $$\begin{aligned} g(\alpha (t))-g(\alpha _0)=\left. {\frac{\partial g}{\partial \alpha }} \right| _{\alpha _0 } (\alpha (t)-\alpha _0 ) \end{aligned}$$

With consideration of Eq. (19),

44 $$\begin{aligned} g(\alpha (t))=\alpha (t+1) \end{aligned}$$

And because $\alpha _0$ is fixed point of $g(\alpha (t))$,

45 $$\begin{aligned} g(\alpha _0 )=\alpha _0 \end{aligned}$$

With (43), (44) and (45) combined,

46 $$\begin{aligned} \alpha (t+1)-\alpha _0 =\left. {\frac{\partial g}{\partial \alpha }} \right| _{\alpha _0 } (\alpha (t)-\alpha _0) \end{aligned}$$

And with replacement $t+1$ in (46),

47 $$\begin{aligned} \alpha (t+2)-\alpha _0 =\left. {\frac{\partial g}{\partial \alpha }} \right| _{\alpha _0 } (\alpha (t+1)-\alpha _0) \end{aligned}$$

With (46) and (47) combined,

48 $$\begin{aligned} \alpha (t+2)-\alpha _0 = \left( \left. \frac{\partial g}{\partial \alpha } \right| _{\alpha _0}\right) ^{2}(\alpha (t)-\alpha _0) \end{aligned}$$

Thus,

49 $$\begin{aligned} \alpha (t+n)-\alpha _0 = \left( \left. {\frac{\partial g}{\partial \alpha }} \right| _{\alpha _0}\right) ^{n}(\alpha (t)-\alpha _0 ) \end{aligned}$$

For stability must be :

50 $$\begin{aligned} \alpha (t+n)=\alpha _0 \end{aligned}$$

This means that

51 $$\begin{aligned} -1<\left. {\frac{\partial g}{\partial \alpha }} \right| _{\alpha _0 } <1 \end{aligned}$$

With derivation of Eq. (19),

52 $$\begin{aligned} \frac{\partial g}{\partial \alpha }=1-B\frac{\partial h}{\partial \alpha } \end{aligned}$$

With (51) and (52) combined,

$$\begin{aligned} 0<B\frac{\partial h}{\partial \alpha }<2 \end{aligned}$$

## Appendix 3

The linear equation of $g_1 (\mathop {\theta _1 }\limits ^{\bullet } (t),\mathop {\theta _2 }\limits ^{\bullet } (t))$ and $g_2 (\mathop {\theta _1 }\limits ^{\bullet } (t),\mathop {\theta _2 }\limits ^{\bullet } (t))$ in $\mathop {\theta _{10} }\limits ^{\bullet }$ and $\mathop {\theta _{20} }\limits ^{\bullet }$,

53 $$\begin{aligned}&g_1 (\mathop {\theta _1 }\limits ^{\bullet } (t),\mathop {\theta _2 }\limits ^{\bullet } (t))-g_1 (\mathop {\theta _{10} }\limits ^{\bullet } ,\mathop {\theta _{20} }\limits ^{\bullet } ) \nonumber \\&\quad = \left. {\frac{\partial g_1 }{\partial \mathop {\theta _1 }\limits ^{\bullet } }} \right| (\mathop {\theta _1 }\limits ^{\bullet } (t)-\mathop {\theta _{10} }\limits ^{\bullet } )+\left. {\frac{\partial g_1 }{\partial \mathop {\theta _2 }\limits ^{\bullet } }} \right| (\mathop {\theta _2 }\limits ^{\bullet } (t)-\mathop {\theta _{20} }\limits ^{\bullet }) \end{aligned}$$

54 $$\begin{aligned}&g_2 (\mathop {\theta _1 }\limits ^{\bullet } (t),\mathop {\theta _2 }\limits ^{\bullet } (t))-g_2 (\mathop {\theta _{10} }\limits ^{\bullet } ,\mathop {\theta _{20} }\limits ^{\bullet } ) \nonumber \\&\quad = \left. {\frac{\partial g_2 }{\partial \mathop {\theta _1 }\limits ^{\bullet } }} \right| (\mathop {\theta _1 }\limits ^{\bullet } (t)-\mathop {\theta _{10} }\limits ^{\bullet } )+\left. {\frac{\partial g_2 }{\partial \mathop {\theta _2 }\limits ^{\bullet } }} \right| (\mathop {\theta _2 }\limits ^{\bullet } (t)-\mathop {\theta _{20} }\limits ^{\bullet } ) \end{aligned}$$

With consideration of Eqs. (27), (28),

55 $$\begin{aligned} g_1 (\mathop {\theta _1 }\limits ^{\bullet } (t),\mathop {\theta _2}\limits ^{\bullet } (t))= & {} \mathop {\theta _1 }\limits ^{\bullet } (t+1) \end{aligned}$$

56 $$\begin{aligned} g_2 (\mathop {\theta _1 }\limits ^{\bullet } (t),\mathop {\theta _2}\limits ^{\bullet } (t))= & {} \mathop {\theta _2 }\limits ^{\bullet } (t+1) \end{aligned}$$

Because $\mathop {\theta _{1_0 } }\limits ^{\bullet }$ and $\mathop {\theta _{2_0 } }\limits ^{\bullet }$ are fixed points of $g_1 (\mathop {\theta _1}\limits ^{\bullet } (t),\mathop {\theta _2 }\limits ^{\bullet }(t))$and $g_2 (\mathop {\theta _1 }\limits ^{\bullet }(t),\mathop {\theta _2 }\limits ^{\bullet } (t))$, respectively,

57 $$\begin{aligned} g_1 (\mathop {\theta _{10} }\limits ^{\bullet } ,\mathop {\theta _{20} }\limits ^{\bullet } )= & {} \mathop {\theta _{10} }\limits ^{\bullet } \end{aligned}$$

58 $$\begin{aligned} g_2 (\mathop {\theta _{10} }\limits ^{\bullet } ,\mathop {\theta _{20} }\limits ^{\bullet } )= & {} \mathop {\theta _{20} }\limits ^{\bullet } \end{aligned}$$

With (53) to (58) combined,

59 $$\begin{aligned} \mathop \theta \limits ^{\bullet }_1 (t+1)-\mathop {\theta _{1_0 } }\limits ^{\bullet }= & {} \frac{\partial g_1 }{\partial \mathop {\theta _1 }\limits ^{\bullet } }\left| {\left( {\mathop {\theta _1 }\limits ^{\bullet } (t)-\mathop {\theta _{1_0 } +\frac{\partial g_1 }{\partial \mathop {\theta _2 }\limits ^{\bullet } }}\limits ^{\bullet } } \right) } \right| \nonumber \\&\times \left( {\mathop {\theta _2 }\limits ^{\bullet } (t)-\mathop {\theta _{2_0}}\limits ^{\bullet } } \right) \end{aligned}$$

60 $$\begin{aligned} \mathop \theta \limits ^{\bullet }_2 (t+1)-\mathop {\theta _{2_0} }\limits ^{\bullet }= & {} \frac{\partial g_2 }{\partial \mathop {\theta _1 }\limits ^{\bullet } }\left| {\left( {\mathop {\theta _1 }\limits ^{\bullet } (t)-\mathop {\theta _{1_0 } +\frac{\partial g_2 }{\partial \mathop {\theta _2 }\limits ^{\bullet } }}\limits ^{\bullet } } \right) } \right| \nonumber \\&\times \left( {\mathop {\theta _2 }\limits ^{\bullet } (t)-\mathop {\theta _{2_0 } }\limits ^{\bullet } } \right) \end{aligned}$$

61 $$\begin{aligned} \left[ {{\begin{array}{l} {\mathop {\theta _1 }\limits ^{\bullet } (t+1)-\theta _{1{ }_0} } \\ {\mathop {\theta _2 }\limits ^{\bullet } (t+1)-\theta _{2_0 } } \\ \end{array} }} \right]= & {} \left[ {{\begin{array}{l} {\frac{\partial g_1 }{\partial \mathop {\theta _1 }\limits ^{\bullet }}} \\ {\frac{\partial g_2 }{\partial \mathop {\theta _1 }\limits ^{\bullet }}} \\ \end{array} }\quad {\begin{array}{l} {\frac{\partial g{ }_1}{\partial \mathop {\theta _2 }\limits ^{\bullet }}} \\ {\frac{\partial g_2 }{\partial \mathop {\theta _2 }\limits ^{\bullet }}} \\ \end{array} }} \right] ^{n}\left[ {{\begin{array}{l} {\mathop {\theta _1 }\limits ^{\bullet } (t)-\mathop {\theta _{1_0 } }\limits ^{\bullet }} \\ {\mathop {\theta _2 }\limits ^{\bullet } (t)-\mathop {\theta _{2_0 } }\limits ^{\bullet }} \\ \end{array} }} \right] \nonumber \\ \end{aligned}$$

And at ($$t+n$$),

62 $$\begin{aligned} \left[ {{\begin{array}{l} {\mathop {\theta _1 }\limits ^{\bullet } (t+n)-\theta _{1{ }_0} } \\ {\mathop {\theta _2 }\limits ^{\bullet } (t+n)-\theta _{2_0 } } \\ \end{array} }} \right] =\left[ {{\begin{array}{l} {\frac{\partial g_1 }{\partial \mathop {\theta _1 }\limits ^{\bullet } }} \\ {\frac{\partial g_2 }{\partial \mathop {\theta _1 }\limits ^{\bullet } }} \\ \end{array} }\quad {\begin{array}{ll} \frac{\partial g_1}{\partial \mathop {\theta _2 }\limits ^{\bullet } } \\ \frac{\partial g_2 }{\partial \mathop {\theta _2 }\limits ^{\bullet } } \\ \end{array} }} \right] ^{n}\left[ {{\begin{array}{l} {\mathop {\theta _1 }\limits ^{\bullet } (t)-\mathop {\theta _{1_0 } }\limits ^{\bullet } } \\ {\mathop {\theta _2 }\limits ^{\bullet } (t)-\mathop {\theta _{2_0 } }\limits ^{\bullet } } \\ \end{array} }} \right] \nonumber \\ \end{aligned}$$

With considering (62),

63 $$\begin{aligned} \left[ {{\begin{array}{ll} {\frac{\partial g_1 }{\partial \mathop {\theta _1 }\limits ^{\bullet } }}&{}\quad {\frac{\partial g_1 }{\partial \mathop {\theta _2 }\limits ^{\bullet } }} \\ {\frac{\partial g_2 }{\partial \mathop {\theta _1 }\limits ^{\bullet } }}&{}\quad {\frac{\partial g_2 }{\partial \mathop {\theta _2 }\limits ^{\bullet } }} \\ \end{array} }} \right] =A \end{aligned}$$

As known, ‘*A*’ is a semi-positive matrix, so

64 $$\begin{aligned} A=\phi ^{T}\Lambda \phi \, \Rightarrow \, A^{n}=\phi ^{T}\Lambda ^{n}\phi \end{aligned}$$

Where $\phi$ Eigen is vector matrix and $\Lambda$ is eigenvalue matrix:

65 $$\begin{aligned} \Lambda =\left[ {{\begin{array}{ll} {\lambda _1} &{} \quad 0 \\ 0&{} \quad {\lambda _2} \\ \end{array}}} \right] \, \Rightarrow \, \Lambda ^{\mathrm{n}}= \left[ {{\begin{array}{ll} {\lambda _1^n } &{} \quad 0 \\ 0 &{} \quad {\lambda _2^n} \\ \end{array}}} \right] \end{aligned}$$

For stability must be:

66 $$\begin{aligned} \left[ {{\begin{array}{l} {\mathop {\theta _1 }\limits ^{\bullet } (t+n)-\theta _{1_0 } } \\ {\mathop {\theta _2 }\limits ^{\bullet } (t+n)-\theta _{2_0 } } \\ \end{array}}} \right] =0\Rightarrow \Lambda ^{n}=0\Rightarrow -1<\lambda _i <1 \end{aligned}$$

Considering Eq. 27 and 28,

67 $$\begin{aligned} \frac{\partial g_1 }{\partial \mathop {\theta _1 }\limits ^{\bullet } }= & {} 1+B_1 \frac{\partial \mathop x\limits ^{\bullet } }{\partial \mathop {\theta _1 }\limits ^{\bullet } } \end{aligned}$$

68 $$\begin{aligned} \frac{\partial g_1 }{\partial \mathop {\theta _2 }\limits ^{\bullet }}= & {} B_1 \frac{\partial \mathop x\limits ^{\bullet } }{\partial \mathop {\theta _2 }\limits ^{\bullet } } \end{aligned}$$

69 $$\begin{aligned} \frac{\partial g_2 }{\partial \mathop {\theta _1 }\limits ^{\bullet }}= & {} B_2 \frac{\partial \mathop y\limits ^{\bullet } }{\partial \mathop {\theta _1 }\limits ^{\bullet } } \end{aligned}$$

70 $$\begin{aligned} \frac{\partial g_2 }{\partial \mathop {\theta _2 }\limits ^{\bullet }}= & {} 1+B_2 \frac{\partial \mathop y\limits ^{\bullet } }{\partial \mathop {\theta _2 }\limits ^{\bullet }} \end{aligned}$$

With combining above equations,

71 $$\begin{aligned} A=\left[ {{\begin{array}{ll} {1+B_1 \frac{\partial \mathop x\limits ^{\bullet } }{\partial \mathop {\theta _1 }\limits ^{\bullet } }} &{} \quad {B_1 \frac{\partial \mathop x\limits ^{\bullet } }{\partial \mathop {\theta _2 }\limits ^{\bullet }}} \\ {B_2 \frac{\partial \mathop y\limits ^{\bullet } }{\partial \mathop {\theta _1 }\limits ^{\bullet }}} &{} \quad {1+B_2 \frac{\partial \mathop y\limits ^{\bullet } }{\partial \mathop {\theta _2 }\limits ^{\bullet }}} \\ \end{array}}} \right] \end{aligned}$$

To calculate eigenvalue of matrix ‘*A*,’

72 $$\begin{aligned}&\left( {1-\lambda +B_1 \frac{\partial \mathop x\limits ^{\bullet } }{\partial \mathop {\theta _1 }\limits ^{\bullet } }} \right) \left( {1-\lambda +B_2 \frac{\partial \mathop y\limits ^{\bullet } }{\partial \mathop {\theta _2 }\limits ^{\bullet } }} \right) \nonumber \\&\quad -B_1 B_2 \frac{\partial \mathop x\limits ^{\bullet } }{\partial \mathop {\theta _2 }\limits ^{\bullet } }\frac{\partial \mathop y\limits ^{\bullet } }{\partial \mathop {\theta _1 }\limits ^{\bullet } }=0 \end{aligned}$$

73 $$\begin{aligned}&\quad \left( {1-\lambda } \right) ^{2}+\left( {B_1 \frac{\partial \mathop x\limits ^{\bullet } }{\partial \mathop {\theta _1 }\limits ^{\bullet } }+B_2 \frac{\partial \mathop y\limits ^{\bullet } }{\partial \mathop {\theta _2 }\limits ^{\bullet } }} \right) \left( {1-\lambda } \right) \nonumber \\&\quad -B_1 B_2 \frac{\partial \mathop x\limits ^{\bullet } }{\partial \mathop {\theta _2 }\limits ^{\bullet } }\frac{\partial \mathop y\limits ^{\bullet } }{\partial \mathop {\theta _1 }\limits ^{\bullet } }=0 \end{aligned}$$

74 $$\begin{aligned}&1-\lambda \nonumber \\&\quad = \frac{-\left( {B_1 \frac{\partial \mathop x\limits ^{\bullet } }{\partial \mathop {\theta _1 }\limits ^{\bullet } }+B_2 \frac{\partial \mathop y\limits ^{\bullet } }{\partial \mathop {\theta _2 }\limits ^{\bullet } }} \right) \pm \sqrt{\left( {B_1 \frac{\partial \mathop x\limits ^{\bullet } }{\partial \mathop {\theta _1 }\limits ^{\bullet } }+B_2 \frac{\partial \mathop y\limits ^{\bullet } }{\partial \mathop {\theta _2 }\limits ^{\bullet } }} \right) ^{2}+4B_1 B_2 \frac{\partial \mathop x\limits ^{\bullet } }{\partial \mathop {\theta _2 }\limits ^{\bullet } }\frac{\partial \mathop y\limits ^{\bullet } }{\partial \mathop {\theta _1 }\limits ^{\bullet } }}}{2}\nonumber \\ \end{aligned}$$

75 $$\begin{aligned} \lambda= & {} 1+\frac{\left( {B_1 \frac{\partial \mathop x\limits ^{\bullet } }{\partial \mathop {\theta _1 }\limits ^{\bullet } }+B_2 \frac{\partial \mathop y\limits ^{\bullet } }{\partial \mathop {\theta _2}\limits ^{\bullet } }} \right) }{2} \nonumber \\&\quad \pm \frac{1}{2}\sqrt{\left( {B_1 \frac{\partial \mathop x\limits ^{\bullet } }{\partial \mathop {\theta _1 }\limits ^{\bullet } }+B_2 \frac{\partial \mathop y\limits ^{\bullet } }{\partial \mathop {\theta _2 }\limits ^{\bullet } }} \right) ^{2}+4B_1 B_2 \frac{\partial \mathop x\limits ^{\bullet } }{\partial \mathop {\theta _2 }\limits ^{\bullet } }\frac{\partial \mathop y\limits ^{\bullet } }{\partial \mathop {\theta _1 }\limits ^{\bullet } }} \end{aligned}$$

With considering the above results and Eq. (66),

76 $$\begin{aligned}&-2<\lambda _i <0 \end{aligned}$$

77 $$\begin{aligned}&\quad -4<\left( {B_1 \frac{\partial \mathop x\limits ^{\bullet } }{\partial \mathop {\theta _1 }\limits ^{\bullet } }+B_2 \frac{\partial \mathop y\limits ^{\bullet } }{\partial \mathop {\theta _2 }\limits ^{\bullet } }} \right) \nonumber \\&\quad \pm \sqrt{\left( {B_1 \frac{\partial \mathop x\limits ^{\bullet }}{\partial \mathop {\theta _1}\limits ^{\bullet } }+B_2 \frac{\partial \mathop y\limits ^{\bullet }}{\partial \mathop {\theta _2 }\limits ^{\bullet } }} \right) ^{2}+4B_1 B_2 \frac{\partial \mathop x\limits ^{\bullet } }{\partial \mathop {\theta _2 }\limits ^{\bullet }}\frac{\partial \mathop y\limits ^{\bullet } }{\partial \mathop {\theta _1}\limits ^{\bullet } }}<0 \end{aligned}$$
